# Hypoxic colorectal cancer cells promote metastasis of normoxic cancer cells depending on IL-8/p65 signaling pathway

**DOI:** 10.1038/s41419-020-02797-z

**Published:** 2020-07-31

**Authors:** Yulong Mi, Lei Mu, Kaiyu Huang, Yibing Hu, Chang Yan, Hui Zhao, Chensen Ma, Xiaolan Li, Deding Tao, Jichao Qin

**Affiliations:** 1 Department of Surgery, Tongji Hospital, Tongji Medical College, Huazhong University of Science and Technology, 430030 Wuhan, China; 2 Molecular Medicine Center, Tongji Hospital, Tongji Medical College, Huazhong University of Science and Technology, 430030 Wuhan, China

**Keywords:** Colorectal cancer, Metastasis

## Abstract

Tumor heterogeneity is an important feature of malignant tumors, and cell subpopulations may positively interact to facilitate tumor progression. Studies have shown that hypoxic cancer cells possess enhanced metastatic capacity. However, it is still unclear whether hypoxic cancer cells may promote the metastasis of normoxic cells, which have greater access to the blood circulation. When cocultured with hypoxic CRC cells or treated with hypoxic CRC cell-derived CM, normoxic CRC cells possessed increased metastatic capacity. Furthermore, hypoxic CRC cell-derived CM was enriched in interleukin 8. Hypoxic CRC cell-derived CM and recombinant human IL-8 both enhanced the metastatic capacity of normoxic cells by increasing the phosphorylation of p65 and then by inducing epithelial-mesenchymal transition. Knockdown of IL-8 in hypoxic CRC cells or the use of an anti-IL-8 antibody attenuated the CM- or rhIL-8-induced prometastatic capacity of normoxic CRC cells. Inhibition or knockdown of p65 abrogated IL-8-induced prometastatic effects. Most importantly, hypoxia-treated xenograft tumors enhanced the metastasis of normoxic CRC cells. Hypoxic CRC cell-derived IL-8 promotes the metastatic capacity of normoxic cells, and novel therapies targeting the positive interactions between hypoxic and normoxic cells should be developed.

## Facts

1. Hypoxic CRC cells are endowed with enhanced metastatic capacity.

2. Hypoxic CRC cells are distant from blood vessels and have reduced access to the blood circulation.

3. Hypoxic CRC cells enhanced the metastatic capacity of normoxic CRC cells via the IL-8/p65 signaling pathway.

4. Hypoxia-treated xenograft CRC tumors promote the metastasis of normoxic CRC cells.

## Introduction

Colorectal cancer (CRC) is the third most common cause of cancer-associated mortality worldwide, and CRC patients typically die due to metastatic lesions^1^. CRC comprises subpopulations of tumor cells that are genotypically or phenotypically divergent. Tumor heterogeneity not only results from tumor cells themselves, but also from different tumor microenvironments, such as those resulting from the maldistribution of blood vessels^2–4^. Notably, within a tumor, there are hypoxic areas where oxygen and nutrients are relatively scarce and normoxic areas where oxygen and nutrients levels are adequate, which may also result in differentces in tumorigenic and metastatic capacity^3,4^.

The tumor vascular network is dynamic^5^, and vascular networks are derived from the formation of new vessels, the modification of existing vessels within tissue, and/or recruitment and differentiation of endothelial precursors from bone marrow, which contribute to vascular heterogeneity^5^. Inadequate function of poorly organized tumor vasculatures results in areas of hypoxia and limited nutrient supply, which can generate distinct microenvironments within the tumor and contribute to inter- and intratumor heterogeneity to ultimately influence the clinical outcome. There is accumulating evidence that, in addition to their mutual interactions, tumor cells also cooperate to promote tumor progression, recurrence, and metastasis^2,6^. Due to the cooperation of tumor cells, increased tumor heterogeneity may lead to a worse prognosis^6^.

Hypoxia can induce tumor cells to develop cancer stem cell-like properties and thus enhance metastasis^3,4^. Tumor cells metastasize by escaping from the primary sites and penetrating through the blood vessels into the circulation. However, hypoxic tumor cells are often distant from blood vessels and may therefore have reduced access to the circulatory system. On the other hand, compared with hypoxic tumor cells, normoxic tumor cells are relatively closer to blood vessels and may possess greater access to the circulation^3,7^. Whether the enhanced metastatic capacity of tumor cells is due to the increased number of hypoxic tumor cells, or hypoxic tumor cells may enhance the metastatic capacity of normoxic tumor cells or both remain largely unknown. Therefore, we hypothesize that hypoxic CRC cells may promote the metastasis of normoxic tumor cells via direct contact and/or in a paracrine manner.

## Materials and methods

### Cell culture

The LoVo and SW48 human colon cancer cell lines were purchased from the cell bank of Chinese Academy of Sciences (Shanghai, China) and cultured in Dulbecco’s modified Eagle’s medium (DMEM; Gibco; Waltham, MA, USA) with 10% fetal bovine serum (FBS; Gibco) and incubated at 37 °C with 5% CO_2_ in a cell culture incubator. XhCRC cells were isolated from human CRC xenograft tumors^8,9^. Details are described in Supplemental Information. Mycoplasma was routinely tested by using mycoplasma detection kit (Thermo Fisher Scientific; Waltham, MA, USA).

### Hypoxic treatment

We introduced two common types of treatment^10,11^. First, cells were cultured in DMEM with 1% FBS in a hypoxic incubator with 5% CO_2_, 94% N_2_ and 1% O_2_. Second, cells were cultured in DMEM with 1% FBS and 300–400 μM CoCl_2_. Refer to Supplemental Information (SI) for details.

### Cell migration and invasion assays

The migration and invasion capacity were determined by wound healing assay and transwell system using transwell inserts (Corning Life Science, Kennebunk, ME, USA)^12,13^. Refer to SI for details.

### In vivo mouse assays and human subjects

All animal studies were performed in the Animal Laboratory Unit of Huazhong University of Science and technology ((HUST) Wuhan, China) under the guidelines and protocols approved by the Institutional Animal Care and Use Committee of Tongji Medical College, HUST (IACUC No.: S652). All human subjects studies were performed under the guidelines and protocols approved by the ethical committe of Tongji Hospital, Tongji Medical College, HUST (IRB ID: 20141106). And informed consent was obtained from all subjects. Refer to SI for details.

### Whole-body imaging assay

In lung metastasis assay, after 8 weeks, the mice were anesthetized by general anesthesia, intraperitoneally injected with 100 μl luciferase substrates (30 mg/ml, Promega), and placed into the imaging dark box platform to get the whole-body image, and the lung metastasis were evaluated by analyzing the photon flux.

### RNA expression analysis

PCR, reverse transcription PCR (RT-PCR) and quantitative real-time PCR (RT-qPCR) analyses were performed as previously described^9,12–14^. Refer to SI for details.

### Immunoblotting, immunofluorescence, and immunohistochemistry

Refer to SI for details.

### Lentiviral transfection

The hypoxia responsive elements (HRE) driving expression of GFP plasmid (p-HRE-GFP) was a gift from Dr. Shideng Bao (Cleveland Clinic, Cleveland, Ohio, USA)^4^. Lentivirus package, Ubi-GFP lentivirus, Ubi-mCherry lentivirus, Ubi-Luciferase lentivirus, and corresponding vector lentivirus were provided by GeneChem Company.

### Inhibition of NFκB signaling pathway

Selective IKK-2 inhibitors (LY2409881 and SC-514)^15,16^, and Dihydroartemisinin (i.e., DHA) and SN50^17,18^ were used.

### Statistical analyses

Statistical significance was calculated with GraphPad Prism 7.0 (GraphPad Software, Inc., San Diego, CA, USA). Data are represented as the mean ± standard deviation, unless otherwise indicated. Experiments were analyzed using an unpaired Student’s *t* test for two groups. Where more than two groups were compared, one-way analysis of variance was used. A value of *P* < 0.05 was considered statistically significant.

## Results

### Hypoxic CRC cells possess higher metastatic capacity than normoxic CRC cells

**Fig. 1:**
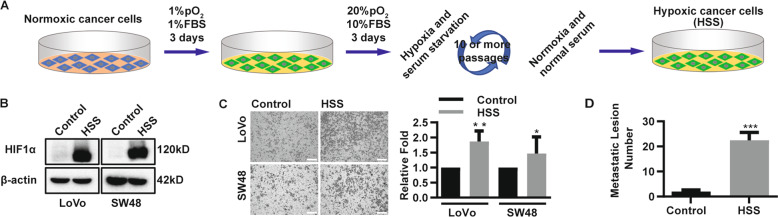
Hypoxic CRC cells possess higher metastatic capacity than normoxic CRC cells. **a** Schematic of the in vitro physical hypoxic treatment of CRC cells. **b** Immunoblot analysis of HIF1α in hypoxic CRC cells. Normoxic CRC cells as control, and β-actin for loading control. **c** Transwell invasion assays. In all, 4 × 10^4^ hypoxic (HSS) and normoxic (Control) CRC cells were incubated, invaded cells were quantified. Scale bars: 200 µm. Mean ± SD from triple experiments. *P < 0.05, **P < 0.01. **d** Quantified numbers of visible metastases in NOD/SCID mice by injecting hypoxic (HSS) and normoxic (Control) xhCRC cells to tail veins (*n* = 5 per group). Data are presented as mean ± SD. ***P < 0.001.

Considering that hypoxic areas have low oxygen and a deficient serum supply, hypoxia in solid tumors is a chronic condition^3,4^. Therefore, to establish chronic hypoxic CRC cells, we cultured CRC cells with low level of oxygen and low serum concentrations (1% oxygen and 1% FBS) instead of normal culture conditions for more than 10 passages (Fig. 1a). In addition, we treated CRC cells with cobalt chloride to induce acute hypoxia. Hence, in describing the experiments, we refer to CRC cells cultured in low oxygen and low serum conditions as hypoxic CRC cells or HSS. Studies have demonstrated that cells in hypoxic environments abundantly express HIF1α^3,19^. Consistent with those of previous studies^10^, our results revealed that the cells abundantly expressed HIF1α (Figs. 1b and S1A). Previous studies have shown that hypoxia alone may promote the metastatic capacity of CRC cells by inducing the expression of matrix metalloproteinase^3^. We found that HSS CRC cells expressed higher mRNA levels of matrix metalloproteinase, such as MMP1, MMP2, and membrane type 1-matrix metalloproteinase 1 (MT1MMP) than normoxic CRC cells (i.e., Control) (Fig. S1B). We then performed Transwell invasion assays and demonstrated that hypoxic CRC cells possessed increased invasive capacity (Fig. 1c). Next, we injected hypoxic and normoxic CRC cells into the tail vein of the NOD/SCID mice. Eight weeks later, hypoxic CRC cells were found to have formed more metastatic lesions than normoxic CRC cells in the lungs of the mice (Fig. 1d). Thus, our findings suggest that hypoxic CRC cells possess high lung metastatic capacity.

### Hypoxic CRC cells enhance the migration, invasion, and metastatic capacity of normoxic CRC cells

**Fig. 2:**
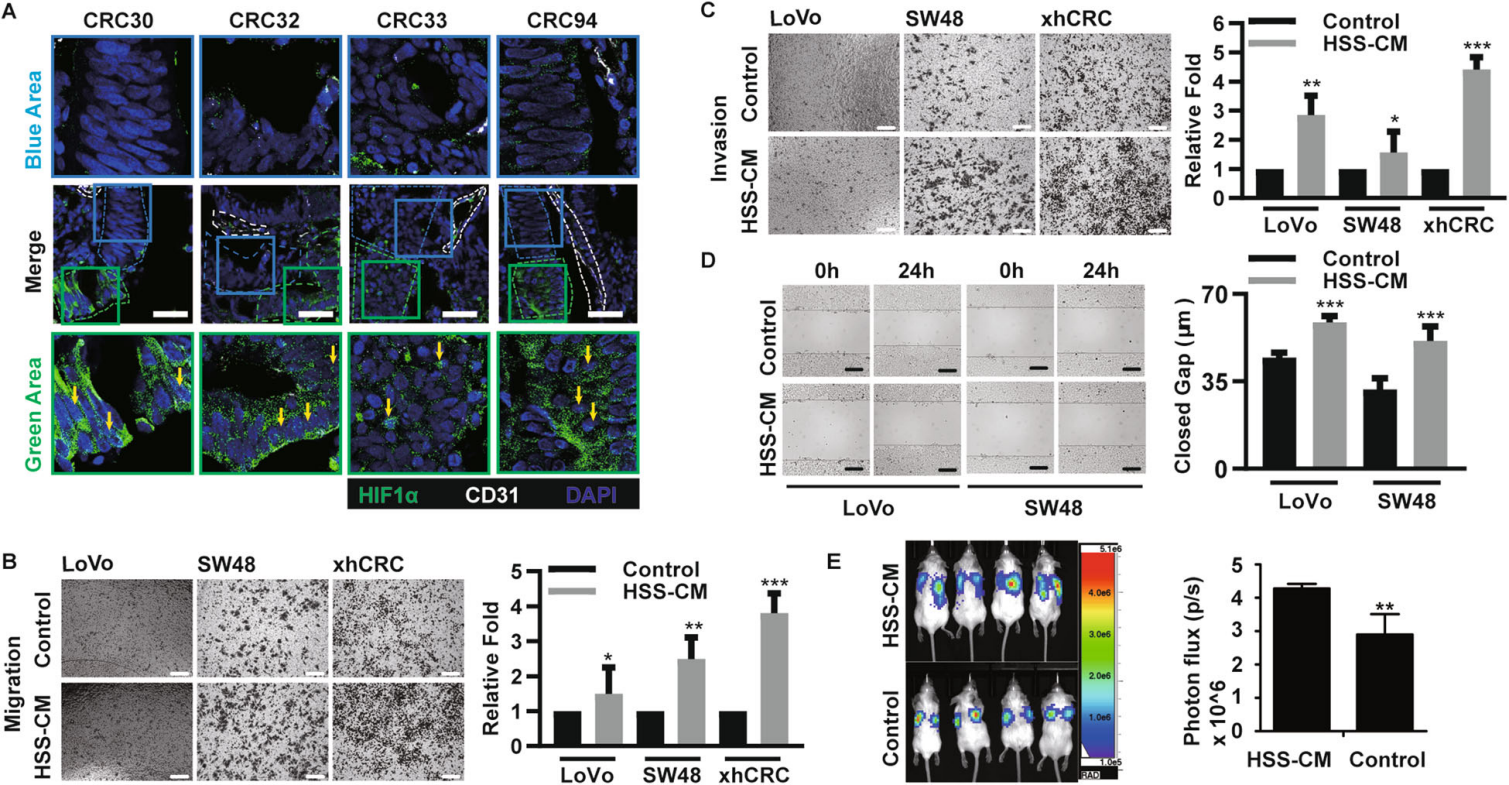
Hypoxic CRC cells enhance the migration, invasion and metastatic capacity of normoxic CRC cells. **a** Immunofluorescence analysis of HIF1α in frozen sections originated from human primary CRC tumors. The white, blue, and green dotted lined area represent for blood vessel, tumor area close to vascular system (i.e., normoxic), and tumor area far from vascular system (i.e., hypoxia), respectively. Yellow arrow represents HIF1α staining inside the nuclei. Scale bar: 50 µm. **b**, **c** Transwell assays. In all, 4 × 10^4^ normoxic CRC cells were cultured in 200 µl control medium or HSS-CM, invaded cells were quantified. Scale bars: 200 µm. Bars represent mean ± SD (*n* = 3). *P < 0.05, **P < 0.01, ***P < 0.001. **d** Wound healing assays. Normoxic CRC cells were cultured in the presence of HSS-CM for 24 h, DMEM/F12 as the control. Scale bars: 200 µm. Bars represent mean ± SD (*n* = 3), ***P < 0.001. **e** In all, 5 × 10^5^ normoxic Luciferase-LoVo cells suspended in 100 µl control medium or HSS-CM were injected into tail vein of NOD/SCID mice (*n* = 4 per group). In all, 100 µl control medium or HSS-CM were given 1 time per 3 days. After 8 weeks, images of the whole-body imaging and quantified analysis are shown. **P < 0.01.

We performed IF and IHC staining of the hypoxic marker protein HIF1α and carbonic anhydrase 9 (CA9)^20^ in sections of human primary CRC tissues and found that the cells expressing increased levels of HIF1α and CA9 were far from the blood vessels; however, the cells expressing decreased levels of HIF1α and CA9 were closer to the blood vessels (Figs. 2a and S2A, B). Therefore, we hypothesized that hypoxic CRC cells might regulate the metastasis of normoxic CRC cells.

To confirm this hypothesis, we first conducted migration and invasion assays of normoxic CRC cells infected with a GFP lentivirus that were mixed hypoxic CRC cells or cultured alone in Transwell chambers. We observed that, when they were cocultured with hypoxic CRC cells, an increased number of normoxic CRC cells penetrated the basement membrane and entered into the lower chamber (Fig. S2C), and an increased number of normoxic CRC cells attached to the lower side of the Transwell chamber (Fig. S2D). Then, we infected normoxic LoVo cells with an mCherry lentivirus vector, and mixed them with normoxic LoVo cells (i.e., N-GFP) or hypoxic LoVo cells (i.e., HSS-GFP) at a ratio of 1:1 and injected them into the tail vein of NOD/SCID mice. Interestingly, an increased number normoxic LoVo-mCherry cells were deposited in the lungs when they were coinjected with hypoxic LoVo cells (Fig. S2E). More importantly, normoxic LoVo cells infected with a luciferase lentivirus vector formed an increased number lung metastatic lesions when they were coinjected with hypoxic LoVo cells (i.e., HSS + N) (Fig. S2F). Together, our results suggest that hypoxic CRC cells promote the lung metastatic capacity of normoxic CRC cells.

### Hypoxic CRC cells enhance the metastatic capacity of normoxic cells via paracrine pathways

To distinguish how hypoxic CRC cells promote the metastatic capacity of normoxic CRC cells, we obtained conditioned medium from hypoxic CRC cells (i.e., HSS-CM), and CM from normoxic CRC cells as the control. Using the above CM, we conducted migration and invasion assays of CRC cells. Notably, HSS-CM significantly increased the migration (Fig. 2b) and invasion capacity (Fig. 2c) of normoxic CRC cells. Consistent with the findings obtained using HSS-CM, cobalt chloride-treated CM (i.e., CoCl_2_-CM) also enhanced the migration and invasion capacity of normoxic CRC cells (Fig. S2G, H). Finally, we performed the wound healing assays. As expected, HSS-CM also enhanced the migratory capacity of normoxic CRC cells (Fig. 2d). It has been previously shown that MMPs promote the metastasis of CRC cells^21^, and we further assessed the expression of MMPs in normoxic CRC cells. We observed that HSS-CM promoted the expression of MMP1, MMP2, and MT1MMP in normoxic CRC cells at the mRNA level (Fig. S2I). Notably, in vivo experiments showed that HSS-CM-treated normoxic CRC cells (LoVo cells) formed larger lung metastatic lesions (Fig. 2e). Together, our data suggest that hypoxic CRC cells may promote the metastatic capacity of normoxic CRC cells through paracrine pathways.

### IL-8 promotes the migratory, invasive, and metastatic capacity of normal CRC cells

**Fig. 3:**
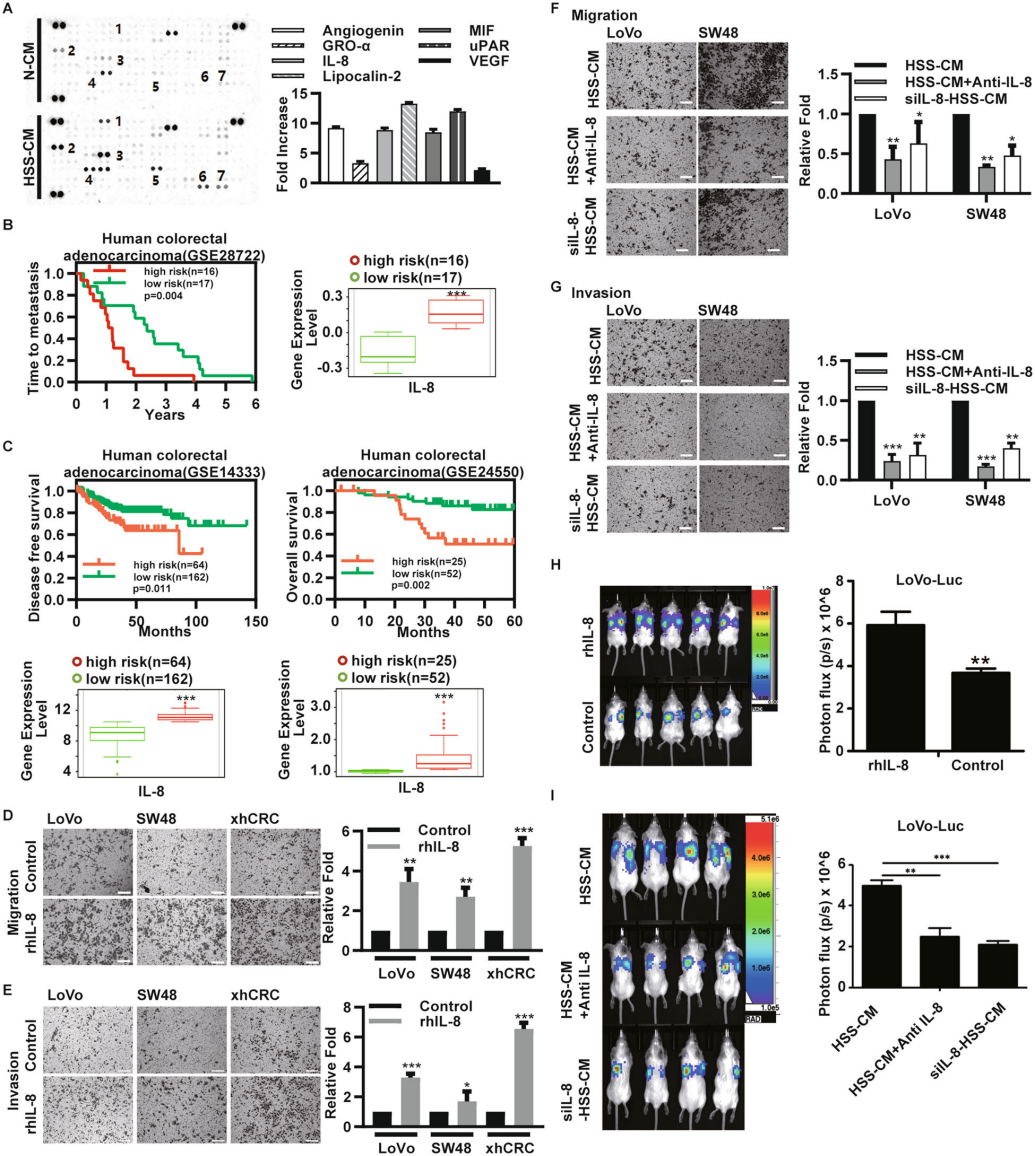
IL-8 promotes the migratory, invasive, and metastatic capacity of normoxic CRC cells. **a** The expression of cytokines in N-CM and HSS-CM derived xhCRC cells. **b** Patients with high expression of IL-8 are tend to metastasis earlier. **c** Kaplan–Meier curves showing that high expression of IL-8 in two microarray data sets are positively associated with poor patient survival. **d**, **e** Transwell assays. In all, 4 × 10^4^ normoxic CRC cells were cultured in 200 µl DMEM with rhIL-8 (2 ng/ml), DMEM only as control, invaded cells were quantified. Scale bars: 200 µm. Bars represent for mean ± SD (*n* = 3). **f**, **g** Transwell assays. In all, 4 × 10^4^ normoxic CRC cells were cultured in 200 µl HSS-CM, HSS-CM plus anti-IL-8 antibody (1 µg/ml), or siIL-8-HSS-CM. Scale bars: 200 µm. Bars represent for mean ± SD (*n* = 3). **h** In vivo metastasis experiment. In all, 5 × 10^5^ normoxic Luciferase-LoVo cells suspended in 100 µl DMEM with rhIL-8 (2 ng/ml) were injected into tail vein of NOD/SCID mice (*n* = 5), and 100 µl DMEM containing rhIL-8 (2 ng/ml) were given every 3 days, DMEM only as control. After 8 weeks, images of the whole-body imaging are shown in the left. Quantified analysis is shown in the right. **i** In vivo metastasis experiment. In all, 5 × 10^5^ normoxic Luciferase-LoVo resuspended in 100 µl HSS-CM, HSS-CM plus anti-IL-8 antibody (1 µg/ml), or siIL-8-HSSCM were injected into tail vein of NOD/SCID mice (*n* = 4). These media were given every 3 days. *P < 0.05, **P < 0.01, ***P < 0.001.

To identify the key factor(s) in HSS-CM that enhance the migratory and invasive capacity of normoxic CRC cells, we evaluated the expression of cytokines in CM derived from the hypoxic xhCRC cells. The cytokine array results indicated that HSS-CM was more enriched in multiple cytokines compared with N-CM (Fig. 3a). Among these cytokines, the expression of interleukin-8 (IL-8) was found to be transcriptionally induced by HIF1α. Studies have shown that IL-8 enhances the migratory, invasive and metastatic capacity through paracrine pathways^22,23^. Therefore, we first focused on the effect of IL-8. According to the microarray data set, GSE28722, metastatic lesions were detected earlier in patients with CRC tumors that highly expressed IL-8 (Fig. 3b). Given the small number of samples, we searched for more microarray data sets and found that the expression of IL-8 in two other microarray data sets was positively associated with poor patient survival (Fig. 3c). Our findings revealed that CM, derived from the CRC cells such as LoVo and SW48 cells treated with CoCl_2_ was also enriched for IL-8 (Fig. S3A).

To determine whether IL-8 promotes the migration and/or invasion of normoxic CRC cells, we conducted the Transwell assays of normoxic CRC cells treated with control medium or medium plus rhIL-8. Consistent with those of previous studies^22,23^, our results demonstrated that rhIL-8 enhanced the migration (Fig. 3d) and invasion (Fig. 3e) of normoxic CRC cells. Then, we performed the migration and invasion assays using HSS-CM supplemented with anti-IL-8 antibody (i.e., HSS-CM + anti-IL-8) and HSS-CM derived cells with knockdown of the expression of IL-8 (i.e., siIL-8-HSS-CM), and the effect of the knockdown of the expression of IL-8 was confirmed (Fig. S3B, C). Consistent with our previous findings, the results showed that siIL-8-HSS-CM or HSS-CM + anti-IL-8 attenuated the pro-invasive and pro–migratory capacity of normoxic CRC cells (Fig. 3f, g).

Then, we injected normoxic LoVo-Luciferase cells into NOD/SCID mice through the tail vein, and the mice were received the injections with DMEM containing rhIL-8 or DMEM only every 3 days until 8 weeks post cell injection. Notably, the normoxic LoVo cells formed more lung metastatic lesions in the mice that received the injection containing rhIL-8 (Fig. 3h). Next, we conducted in vivo experiments using HSS-CM depleted of IL-8, such as siIL-8-HSS-CM or HSS-CM + anti-IL-8. Significantly, depletion of IL-8 in HSS-CM attenuated the lung metastasis-promoting effects on normoxic CRC cells (Fig. 3i). These findings demonstrated that IL-8, one of the most abundant cytokines in the conditioned medium of HSS CRC cells, enhances the metastatic capacity of normoxic CRC cells via paracrine pathways.

### Hypoxic CRC cells promote the metastasis of normoxic CRC cells via IL-8/p65 signaling

**Fig. 4:**
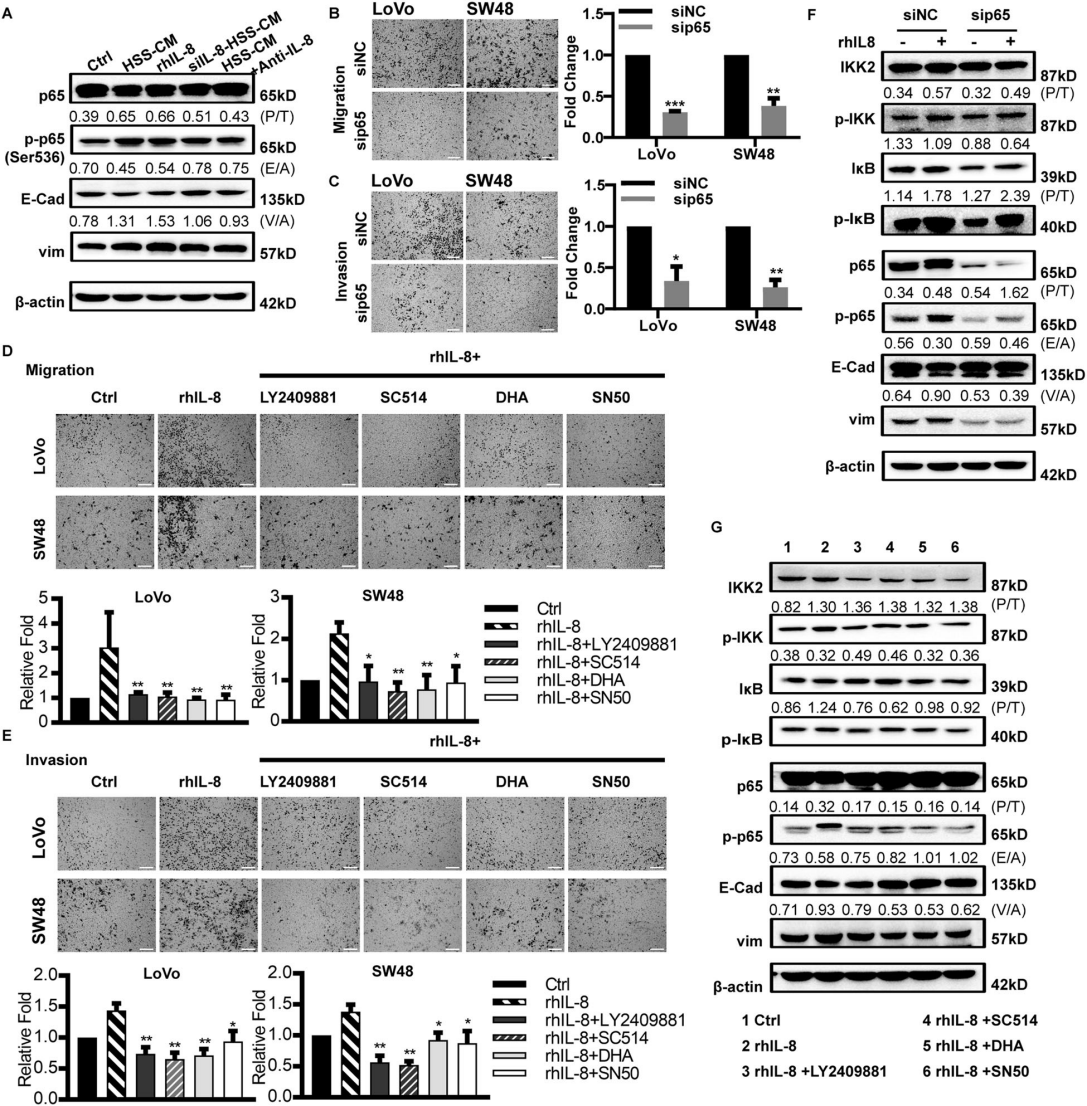
Hypoxic CRC cells promote the metastasis of normoxic CRC cells via IL-8/p65 signaling. **a** Immunoblot analysis of the molecules indicated in normoxic xhCRC cells treated with different media. P/T phosphorylated/total ratio, E/A E-Cad/b-actin ratio, V/A vim/b-actin ratio. **b**, **c** Transwell assays. In all, 4 × 10^4^ normoxic CRC cells infected with siNC or sip65 were cultured in 200 µl DMEM with rhIL-8 (2 ng/ml), invaded cells were quantified. Scale bars: 200 µm. Bars represent for mean ± SD (*n* = 3), *P < 0.05, ** *P* < 0.01, ***P < 0.001. **d**, **e** Transwell assays. In all, 4 × 10^4^ normoxic CRC cells were cultured in different media, such as DMEM (i.e., control), rhIL-8 (2 ng/ml), rhIL-8 plus different inhibitors of NFκB signaling pathway, invaded cells were quantified. Scale bars: 200 µm. Bars represent for mean ± SD (*n* = 3), *P < 0.05, ** *P* < 0.01. **f** Immunoblot analysis of the molecules indicated in normoxic xhCRC cells infected with siNC or sip65 treated with or without rhIL-8. **g** Immunoblot analysis of the molecules indicated in normoxic xhCRC cells treated with different media, such as DMEM (i.e., control), rhIL-8 (2 ng/ml), rhIL-8 plus different inhibitors of NFκB signaling pathway.

Recent studies have shown that IL-8 induces EMT by upregulating the phosphorylation of p65, which is one of five components of the transcription factor nuclear factor kappa-light-chain-enhancer of activated B cells (i.e., NF-kB) pathway, and thus promotes the metastasis of tumor cells^24^. To determine whether hypoxic CRC cell-derived IL-8 phosphorylates p65 and thereby induces EMT, we treated normoxic CRC cells with different media, including HSS-CM, rhIL-8, siIL-8-HSS-CM, and HSS-CM + anti-IL-8. Immunoblotting analysis revealed that enhanced phosphorylation of p65 (according to the phosphorylated/total ratio, P/T ratio) was detected in the normoxic CRC cells treated with the medium enriched in IL-8, such as HSS-CM and rhIL-8, and a decreased P/T ratio was detected when cells were treated with medium depleted of IL-8, such as siIL-8-HSS-CM and HSS-CM + anti-IL-8 (Fig. 4a). Notably, CRC cells expressed less E-cadherin (decreased E-cadherin/b-actin ratio, E/A ratio) and more Vimentin (increased Vimentin/b-actin ratio, V/A ratio) when treated with the medium enriched in IL-8, and in the contrast when treated with the medium depleted of IL-8 (Fig. 4a).

To further confirm the above results, we knocked down p65 in the CRC cells. As shown in Fig. S4A, B, in the CRC cells such as LoVo and SW48 cells, the expression of p65 was significantly downregulated at the mRNA and protein levels upon administration of p65 siRNA. More importantly, the rhIL-8-treated CRC cells exhibited decreased migration (Fig. 4b) and invasion capacity (Fig. 4c) in the Transwell assays and migrated more slowly in the wound healing assays (Fig. S4C) when p65 was knocked down. To further determine whether p65 mediates the metastasis of normoxic CRC cells induced by HSS CRC cell-derived IL-8, we applied chemicals that specifically inhibit p65, including LY2409881 and SC-514^15,16^, and dihydroartemisinin (i.e., DHA) and SN50^17,18^. Consistent with the findings of obtained from knocking down p65, the results demonstrated that, in the presence of the above inhibitors, the rhIL-8-treated CRC cells possessed the decreased migratory (Figs. 4d and S4D) and invasive capacity (Fig. 4e). Taken together, our findings suggest that the hypoxic CRC cells up-regulate the migration and invasion of normoxic CRC cells through the IL-8/p65 signaling pathway.

To explore whether HSS CRC cell-derived IL-8 promotes the metastasis of normoxic CRC cells by activating the p65 signaling pathway, thus promoting EMT, we performed immunoblotting assays to detect p65 knockdown in normoxic CRC cells with or without rhIL-8 treatment. As shown in Fig. 4f, the enhanced phosphorylation of IKK2, IkB and p65 was observed in the normoxic CRC cells treated with rhIL-8, and the expression of E-cadherin was increased while that of Vimentin decreased. Notably, upon knockdown of p65, the phosphorylation of IKK and IkB was increased, but the expression of E-cadherin and Vimentin was not changed which was similar to the results observed in the siNC group, suggesting that IL-8 may mediate EMT through IKK2/IkB/p65 signaling.

Then, we applied the inhibitors of the IKK2/IkB/p65 signaling pathway to normal xhCRC cells and then performed immunoblotting analysis. As demonstrated in Fig. 4g, the decreased phosphorylation of p65 was observed in the normoxic CRC cells treated with the inhibitors of p65; furthermore, the expression of E-cadherin was increased, while that of Vimentin was decreased in the normoxic CRC cells treated with inhibitors.

Our findings demonstrate that hypoxic CRC cell-derived IL-8 promotes the EMT of normoxic CRC cells through p65 signaling pathways and thus promotes the metastasis of normoxic CRC cells.

### Hypoxia-treated CRC tumor show increased the expression of IL-8

To better detect the hypoxia level of tumors, and considering that HIF1α is easily degraded, we infected xhCRC cells with a lentivirus vector for which the HRE drive the expression of GFP (HRE-GFP) at a multiplicity of infection (MOI) of 25^4^. Next, we performed FACS and immunofluorescence assays of the CRC cells infected with the HRE-GFP vector. Our data showed that hypoxia-treated xhCRC cells highly expressed GFP while only a few cells expressed GFP under normal culture conditions (Fig. S5B, C), suggesting that the HRE-GFP vector faithfully reports the hypoxic state in the hypoxic CRC cells.

**Fig. 5:**
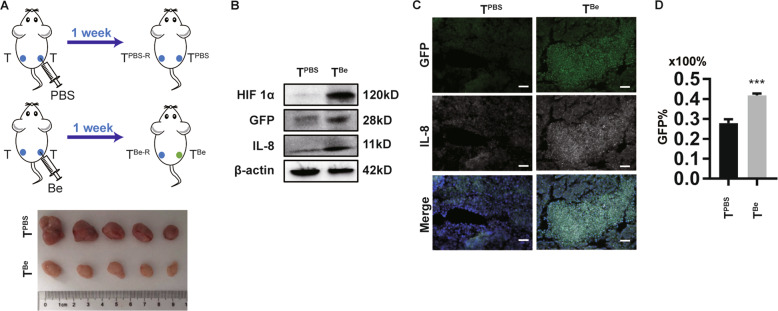
Hypoxia-treated CRC tumors show increased expression of IL-8. **a** Schematic of Hypoxia-induced CRC tumor. In all, 1 × 10^6^ xhCRC-HRE-GFP cells were injected bilaterally into flanks of NOD/SCID mice (*n* = 5). After the tumor size was up to ~ 30 mm^3^, Bevacizumab (Be, 5 mg/kg body weight) was administered per 3 days by intratumor injections. Animals received injection of PBS as control. The tumors injected with Bevacizumab or PBS are referred to T^Be^ and T^PBS^, and another side tumor in each group are referred to T^Be-R^ and T^PBS-R^, respectively. **b** Immunoblot analysis of the molecules indicated in T^PBS^ and T^Be^. **c** Freezing sections of T^PBS^ and T^Be^ were stained for the molecules indicated. Scale bar: 50 µm. **d** Quantified analysis of GFP^+^ xhCRC-HRE-GFP cells in tumors indicated by FACS. Bars represent for mean ± SD (*n* = 3). ***P < 0.001. n.s. no significance.

Anti-angiogenic drugs such as bevacizumab (i.e., Be) affect the formation and function of the tumor-related vascular system; they also cause oxygen and nutrient supply shortages^25,26^. To obtain hypoxic tumors in the xenograft model, Be-induced tumor hypoxia of CRC xenograft tumors was utilized (Fig. 5a upper panel). The tumors were harvested one week later. Interestingly, Be-treated tumors (i.e., T^Be^) were much redder than those treated with PBS (i.e., T^PBS^), implying that bevacizumab-treated tumors possessed a decreased blood supply (Fig. 5a under panel). More interestingly, upon administration of bevacizumab via intratumoral injection, the tumor cells highly expressed GFP, HIF1α and IL-8 (Fig. 5b, c); and furthermore, a greater number of cells were GFP^+^ cells (Figs. 5d and S5D), suggesting that T^Be^ were more hypoxic than T^PBS^. Consistent with the findings obtained from the xenograft model, the immunofluorescence assay of human primary CRC tumors also showed that hypoxic CRC cells expressed a higher level of IL-8 than normoxic tumor cells (Fig. S5E).

Our data showed that bevacizumab treatment reliably induced tumor hypoxia and serum starvation and that hypoxia-treated CRC tumors expressed increased IL-8.

### Hypoxia-treated CRC tumors promote the metastasis of normoxic CRC cells

**Fig. 6:**
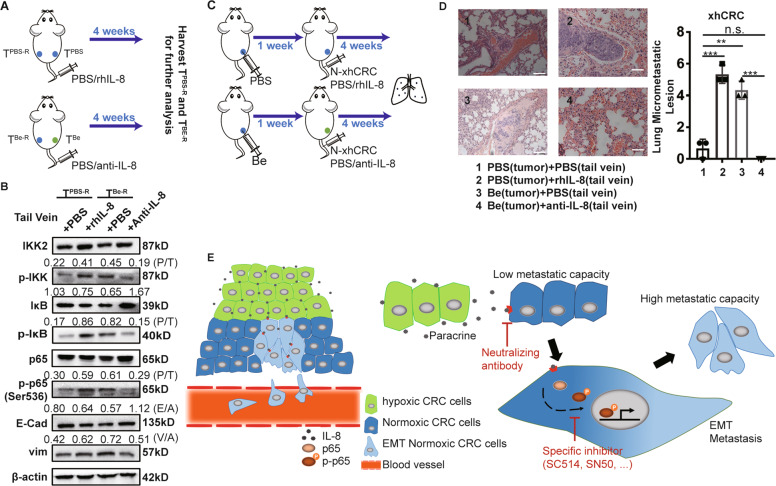
Hypoxia-treated CRC tumors promote the metastasis of normoxic CRC cells. **a** Schematic of hypoxic tumor impacting normoxic CRC cells or tumors. The mice bearing tumors referred as T^Be-R^ and T^PBS-R^ were further injected PBS, rhIL-8 and anti-IL-8, respectively, after 4 weeks, T^Be-R^ and T^PBS-R^ were harvested for further analysis. **b** Immunoblot analysis of the molecules indicated in T^PBS-R^ and T^Be-R^ originated from A. P/T phosphorylated/total ratio, E/A E-Cad/b-actin ratio, V/A vim/b-actin ratio. **c** Schematic of lung metastasis. In all, 1 × 10^6^ xhCRC-HRE-GFP cells were injected unilaterally into flanks of NOD/SCID mice (*n* = 5). After the tumor size was up to ~30 mm^3^, Bevacizumab (Be, 5 mg/kg body weight) was administered per 3 days by intratumor injections, the mice received injection of PBS as control. And 1 week later, normoxic 1 × 10^6^ xhCRC cells suspended in 100 µl PBS only or plus rhIL-8 or plus anti-IL-8 were injected into tail vein and the above media were injected once per 3 days. After 4 weeks, Lungs were harvested. **d** Representative image and quantified analysis of lung metastatic lesions are shown. Scale bar: 100 µm. **P < 0.01, ***P < 0.001. n.s. no significance. **e** Schematic of the proposed model. Normoxic CRC cells are closer to the blood vessel than hypoxic CRC cells. Hypoxic CRC cells promote the metastasis of normoxic CRC cells by secreting IL-8, followed by hyper-phosphorylating p65 and then inducing EMT of normoxic CRC cells.

To determine whether hypoxic tumor cells promote the EMT of normoxic tumor cells and thus enhance metastasis via IL-8/p65 axis signaling in our hypoxia-treated tumor model, we performed immunoblotting analysis of the tumors originating from the mice, which were treated as shown in Fig. 6a. Our results demonstrated that compared with PBS-treated tumors injected with PBS via the tail vein, Be-treated tumors injected with PBS or PBS-treated tumors injected with rhIL-8 showed greater phosphorylation of p65, increased Vimentin and decreased E-cadherin, and more importantly, the anti-IL-8 antibody abolished this effect in Be-treated tumors (Fig. 6b).

Then, we injected bevacizumab into established xenograft CRC tumors in female NOD/SCID mice and injected PBS as a control, and we examined the lung metastatic potential of normoxic xhCRC cells by injecting them into the tail vein of the mice. In addition, medium containing anti-IL-8 antibody, rhIL-8 or PBS as control medium was injected into the tail vein of the mice (Fig. 6c). As shown in Fig. 6d, hypoxic tumors induced normoxic CRC cells to establish a greater number of micrometastases in the lungs, and anti-IL-8 antibody abolished this effect. These results suggest that hypoxia-treated tumors promote the metastasis of normoxic CRC cells via IL-8.

## Discussion

Hypoxia occurs frequently in human cancers, particularly solid tumors such as CRCs. Due to rapid growth, solid tumors will suffer from hypoxia owing to the inability of the local vasculature to supply enough oxygen and nutrients^27,28^. Over the past few decades, numerous studies have indicated that hypoxia promotes tumor progression by enhancing tumor angiogenesis and metastasis^3,4,10,19,27,28^. In CRC, Cannito et al^29^. showed that hypoxia-induced ROS induced early EMT-related events. In addition, HIF1α can promote metastasis by inducing extracellular matrix remodeling^30^ and interacting with β-catenin and Nur77^31^. Consistent with previous studies focusing on hypoxia only, our findings indicated that at least two widely used CRC cell lines (i.e., LoVo and SW48) show enhanced metastatic capacity upon exposure to hypoxic serum starvation (Fig. 1).

It is widely accepted that tumors display intratumoral heterogeneity^2,32,33^. Among the multiple factors of the tumor microenvironment, the blood and/or nutrient supply are critical in inducing tumor heterogeneity^33,34^. The heterogeneity of hypoxia is mainly rooted in the following two aspects: uncontrolled tumor cell growth and the immaturity of the tumor vasculature. Aberrant blood vessel formation cannot keep up with rapid cell division, which causes a shortage in the supply of oxygen and nutrients^27,35,36^. In this study, we performed IF and IHC on quite a few CRC tumor surgical samples and found that normoxic and hypoxic cell subpopulations simultaneously exist in CRCs (Figs. 2a and S2A, B).

The limited availability of oxygen and nutrients should constrain clonal expansion within tumors, which may lead to competitive interactions^36^. Meanwhile, studies have shown that cooperation between different subpopulations promotes tumor progression^37–39^. HIF signaling may enhance the metastatic capacity of hypoxic cancer cells. However, the absence of a microvasculature reduces the opportunity for metastasis through the vascular system. Therefore, in addition to possessing high metastatic capacity, hypoxic tumor cells may also promote the metastasis of normoxic cells. Our findings showed that normoxic CRC cells, upon coculture or coinjection with hypoxic CRC cells and treatment with hypoxic CRC cell-derived CM, possess enhanced migratory, invasive and metastatic capacity (Figs. 2 and S2).

Although in vitro hypoxic models are widely used in cellular hypoxia studies, these models have disadvantages. First, traditional hypoxia is induced for 3–24 h^4,10,11,40^, which are acute hypoxic conditions. Furthermore, tumor hypoxia in vivo is complicated^34^. However, limiting the oxygen density in a specific range may not fully simulate the heterogeneous oxygen density in vivo. Third, toxicity caused by CoCl_2_ cannot be entirely excluded^41^. Finally, tumor cells are subject to a shortage supplying of oxygen and nutrients, and hypoxia alone is not sufficient. Accordingly, in the present research, we initially used 10 cycles of hypoxic serum starvation and recovery culture to obtain the hypoxic-CRC cells (Fig. 1a). The phenotype (enhanced metastatic ability) was maintained for a period even when cells were isolated from the hypoxic and nutrient-starved environment. In addition, in a study of OSCC, Li et al.^40^ injected exosomes into the centers of the xenograft tumors, but there is a lack of solid and direct in vivo data to illustrate the communication between hypoxic and normoxic cancer cells. Anti-angiogenic drugs such as bevacizumab can cause oxygen and nutrient supply shortages, and bevacizumab-induced hypoxia of tumors has been reported in breast cancer patients^25^ and cervical carcinoma-bearing mice^26^ treated with bevacizumab. Such a bevacizumab-treated tumor hypoxia model is mainly used to investigate the characteristics of hypoxic tumor cells themselves or the mechanism of bevacizumab resistance through intravenous or intraperitoneal injection^25,26^. The utilization of a bevacizumab-treated tumor hypoxia model in the research on the communication between hypoxic and normoxic cancer cells has not been reported. In this study, we utilized bevacizumab to induce tumor hypoxia in CRC xenograft tumors through intratumoral injection (Fig. 5a–d). Hence, we successfully obtained hypoxic and normoxic tumors that were separated spatially but that interacted functionally in one mouse. More importantly, using an HRE-drivien GFP lentivirus vector, hypoxic conditions in the cells are easily monitored. We maintain that hypoxia-treated tumors are a reliable model to study the behavior of hypoxic tumors, especially the communication between hypoxic tumor clones and normoxic ones, particularly, in vivo.

In this study, we found that IL-8 was enriched in conditioned medium derived from hypoxic CRC cells (Fig. 3a), and hypoxia-treated tumors expressed much higher levels of IL-8 than normoxic tumors (Fig. 5b, c), which is in line with our observations of human primary CRC tumors (Fig. S5E). IL-8 plays an important role in tumor progression^23^, especially in the field of tumor metastasis and in nasopharyngeal carcinoma^22^, glioma^24^, hepatocellular cancer^42^, thyroid cancer^43^, and CRC^44^. In the process of hypoxia, hyperregulated HIF1α may transcriptionally induce the expression of a variety of genes, including IL-8^23^. The sources of IL-8 in the tumor site are diverse and include autocrine sources^24,45^ or paracrine sources derived from senescent carcinoma-associated fibroblasts^46^ and tumor associated macrophages^47^. Our research showed that hypoxic CRC cell-derived IL-8 promoted the metastatic capacity of normoxic CRC cells (Fig. 3). More importantly, in the xenograft model, hypoxia-treated tumors promoted the metastasis of normoxic CRC cells via IL-8 (Fig. 6). In the present study, we found that IL-8 induced the phosphorylation of IKK2, IκB, and p65 and promoted EMT of normoxic CRC cells (Figs. 4a and 6b), and the inhibition of p65 attenuated the effects of IL-8 on normoxic CRC cells (Fig. 4b–g).

We provide evidence that hypoxic cancer cells, in addition to possessing enhanced metastatic capacity, can also promote the lung metastasis of normoxic cells by secreting IL-8 (Fig. 6e). Certainly, the result of spontaneous metastasis models or other metastasis models can provide more solid evidence, however this phenomenon truly provides a new perspective for the treatment of tumor metastasis, that is, in addition to targeting hypoxic tumor cells, it is also necessary to target the pathway that mediates the promotion of the metastasis of normoxic CRC cells by hypoxic cells.

## Supplementary information

Supplementary Figure S1

Supplementary Figure S2

Supplementary Figure S3

Supplementary Figure S4

Supplementary figure S5

Supplementary figure legends

Supplementary Information
