# Supplementary figures and images for: Hypoxic colorectal cancer cells promote metastasis of normoxic cancer cells depending on IL-8/p65 signaling pathway
Source: Cell Death Dis. 2020 Jul 31;11(7):610. doi: 10.1038/s41419-020-02797-z (PMC7395770; doi:10.1038/s41419-020-02797-z)

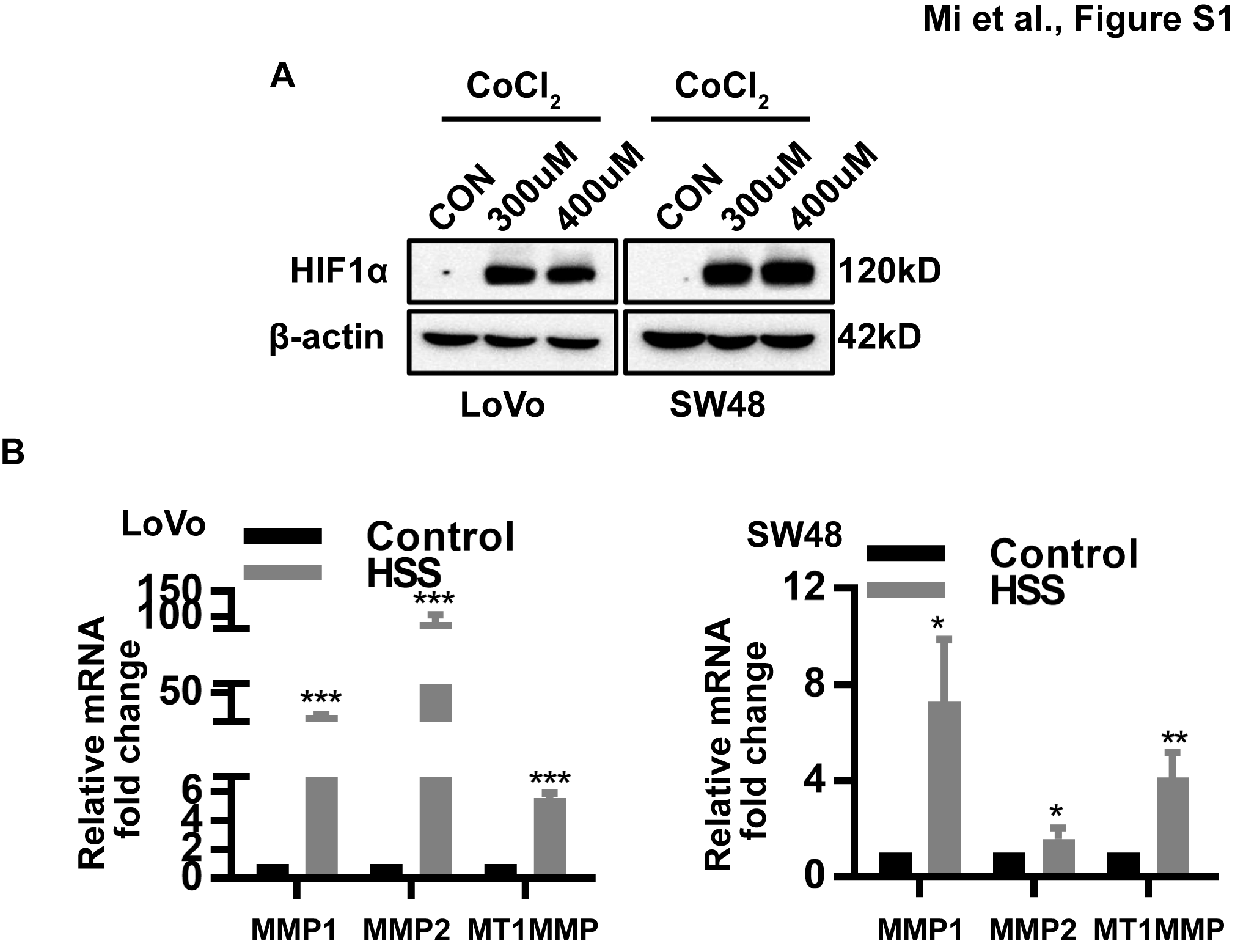

Supplement: Supplementary file 1 — Supplementary Figure S1 [file 41419_2020_2797_MOESM1_ESM.tif]

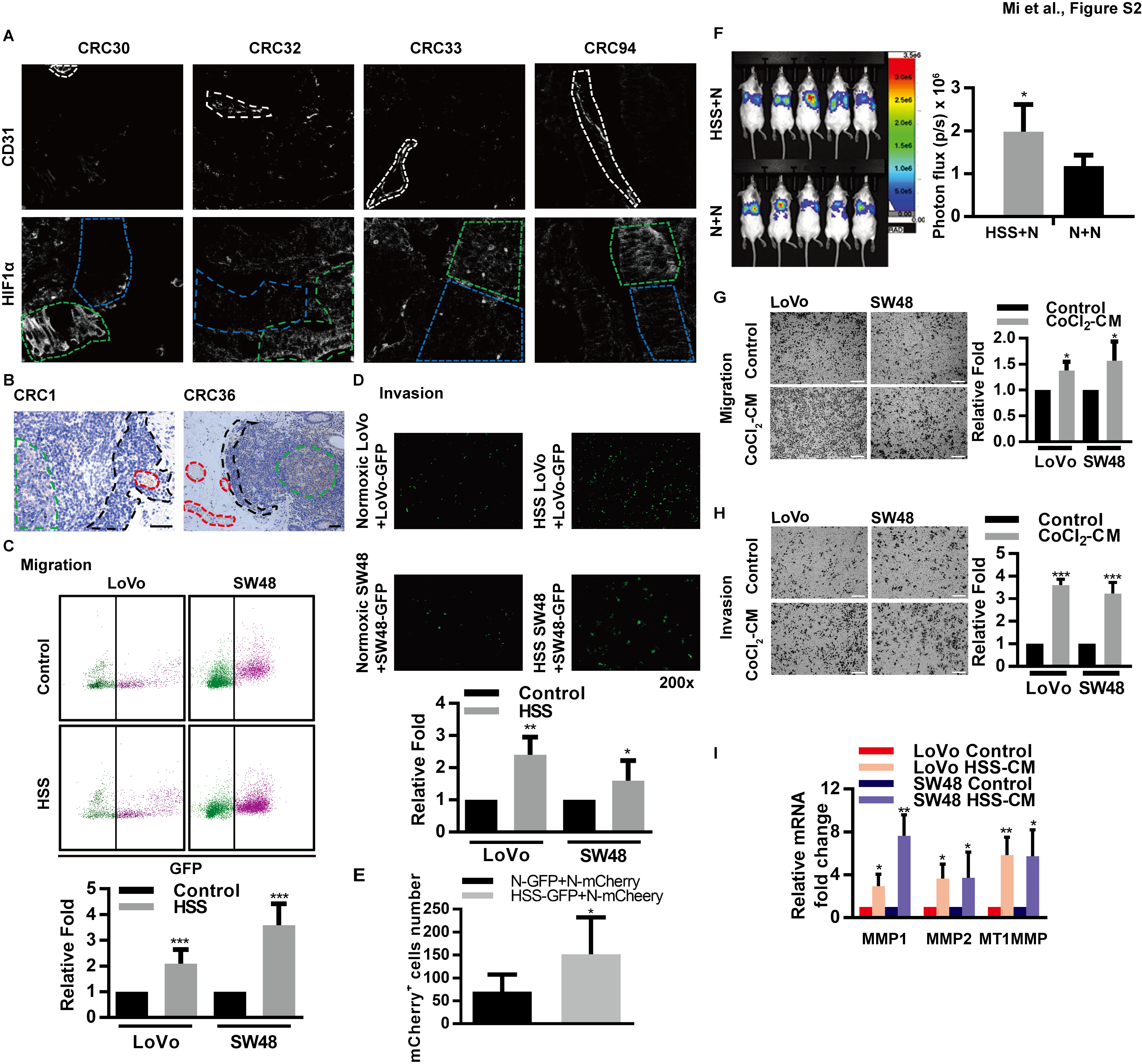

Supplement: Supplementary file 2 — Supplementary Figure S2 [file 41419_2020_2797_MOESM2_ESM.tif]

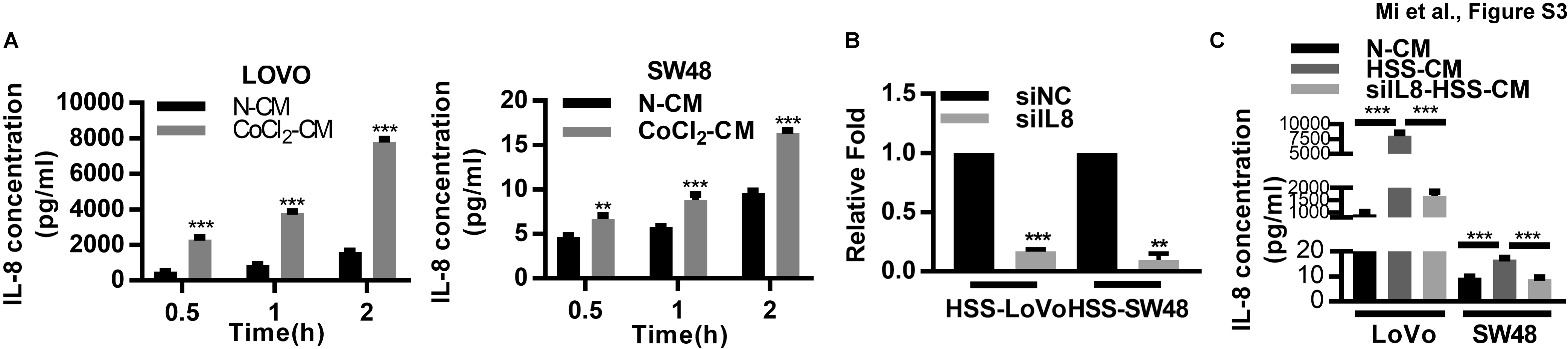

Supplement: Supplementary file 3 — Supplementary Figure S3 [file 41419_2020_2797_MOESM3_ESM.tif]

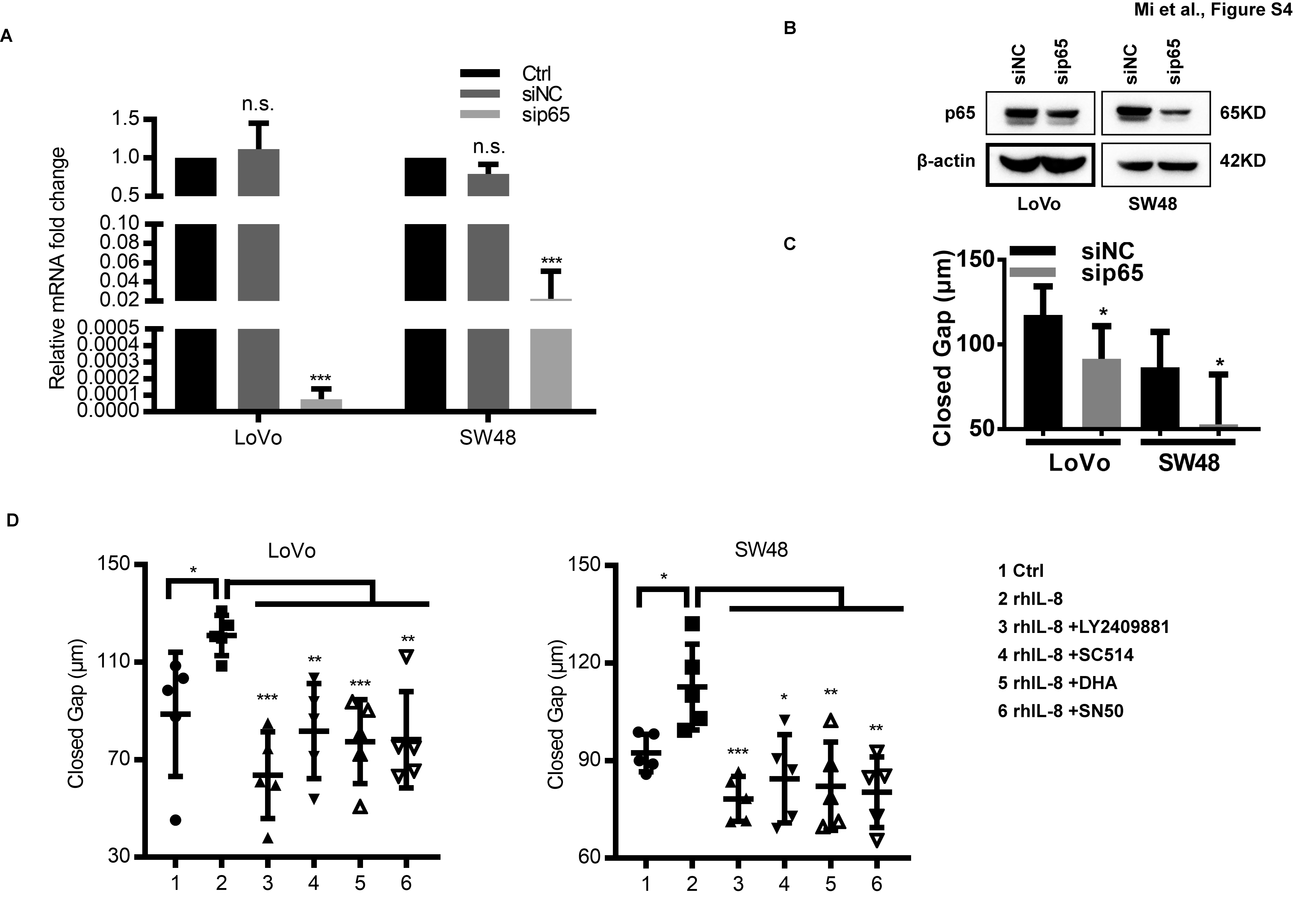

Supplement: Supplementary file 4 — Supplementary Figure S4 [file 41419_2020_2797_MOESM4_ESM.tif]

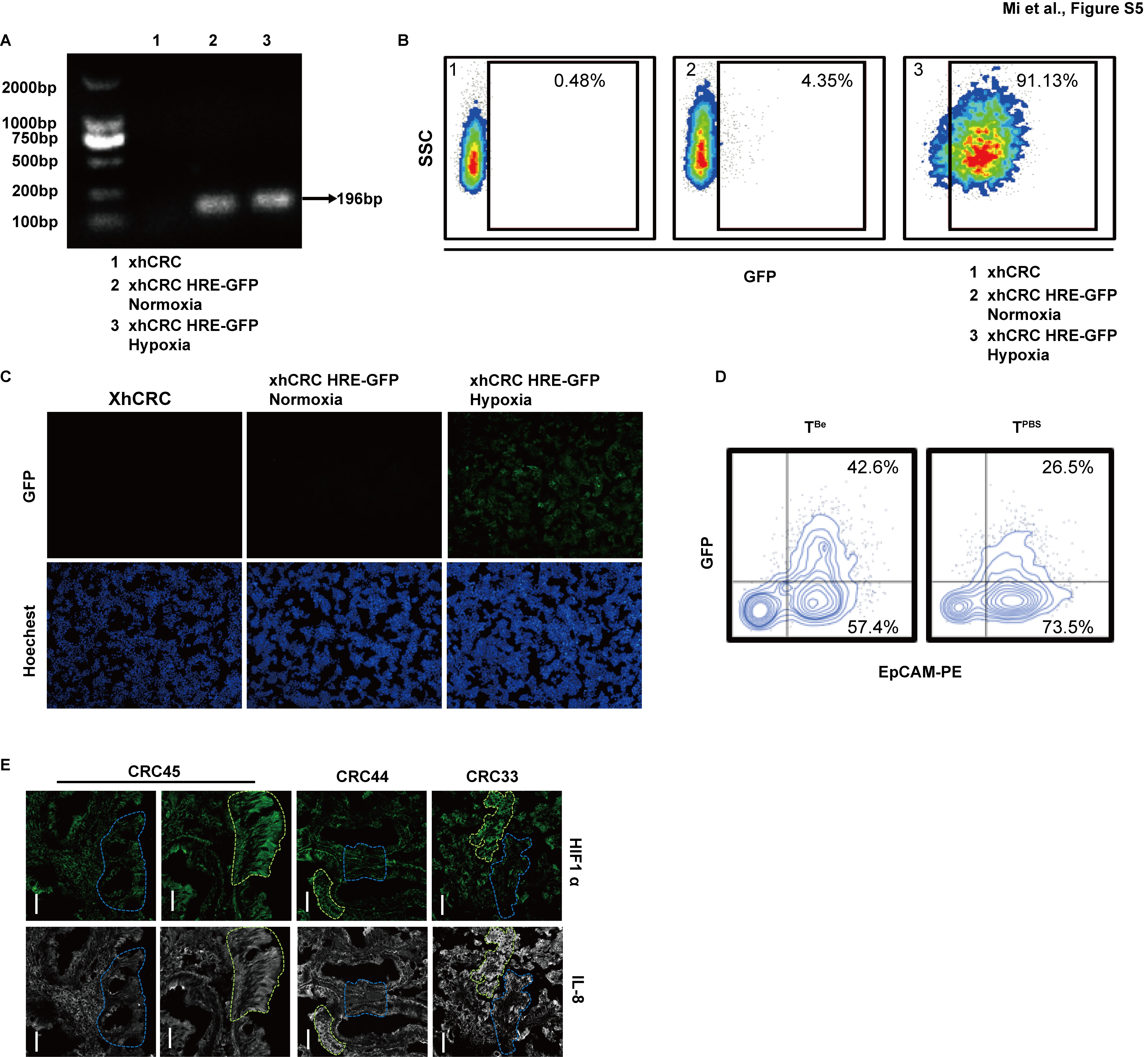

Supplement: Supplementary file 5 — Supplementary figure S5 [file 41419_2020_2797_MOESM5_ESM.tif]
