# Supplementary material for: Hypoxic colorectal cancer cells promote metastasis of normoxic cancer cells depending on IL-8/p65 signaling pathway
Source: Cell Death Dis. 2020 Jul 31;11(7):610. doi: 10.1038/s41419-020-02797-z (PMC7395770; doi:10.1038/s41419-020-02797-z)
Supplement: Supplementary file 6 — Supplementary figure legends [file 41419_2020_2797_MOESM6_ESM.docx]

Supplementary Figures Legends for

Hypoxic colorectal cancer cells promote metastasis of normoxic cancer cells depending on IL-8/p65 signaling pathway

Yulong Mi1,2*, Lei Mu1,2*, Kaiyu Huang1,2, Yibing Hu1,2, Chang Yan1,2, Hui Zhao2, Chensen Ma1,2, Xiaolan Li2, Deding Tao2, Jichao Qin1,2^#^

*These authors contributed equally to this work.

^#^ Jichao Qin, MD, PhD

Email: [jcqin@tjh.tjmu.edu.cn](mailto:jcqin@tjh.tjmu.edu.cn)

**Supplementary Figures Legends**

**Fig. S1. (A)** Immunoblot analysis of HIF1α in CoCl_2_-induced CRC cells. Normoxic CRC cells as control, and β-actin for loading control. **(B)** Real time PCR analysis of MMP1, MMP2 and MT1MMP mRNA expression in normoxic (Control) and hypoxic (HSS) CRC cells such as LoVo and SW48 cells. *P < 0.05, ** P < 0.01, ***P < 0.001.

**Fig. S2. (A)** Refer to Fig 2A. **(B)** Immunohistochemistry analysis of carbonic anhydrase 9 (CA9) in human primary CRC tumors. The red, black, and green dotted lined area represent for blood vessel, tumor area close to (i.e., normoxia) or far from (i.e., hypoxia) vascular system, respectively. Scale bar: 100µm. **(C)** and **(D)** Transwell assays. Transwell assays. 4x10^4^ GFP^+^-normoxic CRC cells were co-cultured with 4x10^4^ normoxic CRC cells (Control) or 4x10^4^ hypoxic CRC cells (HSS) in the upper chamber, GFP^+^ CRC cells in the lower chamber were quantified. Bars represent mean ± SD (n=3), *P < 0.05, **P < 0.01, ***P < 0.001. **(E)** Tumor cells deposit assay in vivo. 5x10^5^ normoxic mCherry^+^-LoVo mixed with equal amount of normoxic GFP^+^-LoVo or hypoxic GFP^+^-LoVo were injected into tail vein of NOD/SCID mice (n=5 per group). After 24 hours, mice were sacrificed, and lung were harvested. The mCherry^+^-LoVo cells in the freezing sections of lung were quantified. Quantified analysis is shown. *P < 0.05. **(F)** In vivo metastasis assay. 5x10^5^ normoxic Luciferase^+^-LoVo mixed with equal amount of normoxic LoVo or hypoxic LoVo were injected into tail vein of NOD/SCID mice (n=5 per group). After 8 weeks, 100 µl D-Luciferin (30mg/ml) were injected into abdominal cavity of anesthetic mice. Images of the whole-body imaging in in vivo metastasis assay are shown. *P < 0.05. **(G)** and **(H)** Transwell assays. 4x10^4^ normoxic CRC cells were cultured in 200µl control medium or CoCl_2_-CM. Scale bars: 200µm. **(I)** Real time PCR analysis of the mRNA indicated in normoxic CRC cells cultured in control medium or HSS-CM for 24 hours. *P < 0.05, ** P < 0.01

**Fig. S3. (A)** The expression of IL-8 in CoCl_2_-CM through ELISA assays. The conditioned media (i.e., CoCl_2_-CM) were collected at the different time (0.5, 1, and 2 h) post the washout of CoCl_2_. Quantified analysis is shown. Mean ± SD from triple experiments. **P < 0.01, ***P < 0.001. **(B)** The knock down of IL8 in HSS CRC cells by siRNA. Knockdown of IL-8 in mRNA level was measured by Real Time PCR. Mean ± SD from triple experiments. **(C)** The expression of IL-8 in HSS-CM through ELISA assays. Mean ± SD from triple experiments.

**Fig. S4** **(A)** The knock down of p65 in N-CRC cells by SiRNA. The expression of p65 in N-CRC cells was measured by real time PCR at the levels of mRNA upon knockdown of p65. Quantified analyses are shown. ***P < 0.001. **(B)** Representative immunoblot analysis of total p65 in normal LoVo and SW48 cells infected with siNC or sip65. β-actin was loading control. **(C)** Wound healing assays. Normoxic CRC cells infected with siNC or sip65 were cultured in the DMEM with rhIL-8 (2 ng/ml) for 24 h. Bars represent for mean ± SD (n=3), **P* < 0.05. **(D)** Wound healing assays. Normoxic LoVo and SW48 cells were cultured in the different media, such as DMEM, DMEM with rhIL-8 (2ng/ml), DMEM with rhIL-8 supplemented with different inhibitors of NFκB signaling pathway (LY2409881 1 uM, SC-514 10 uM, DHA 8 uM, SN50 18 uM) for 24 h. Quantified analysis are shown. Mean ± SD from triple experiments. *P < 0.05, ** P < 0.01, ***P < 0.001.

**Fig. S5 (A)** PCR detection of GFP sequence in genomic DNA of normoxic and hypoxic xhCRC-HRE-GFP cells, and uninfected cells as a negative control. **(B)** and **(C)** The GFP+ cells were measured by flow cytometry **(B)** and IF **(C). (D)** Representative FACS files for Quantified analysis of the GFP^+^ cells in the xhCRC-HRE-GFP cell-derived tumors such as T^Be^, T^PBS^. The cells for positive in EpCAM staining are epithelial cells (i.e., xhCRC cells). **(E)** Freezing sections of human primary CRC tissues were stained for the molecules indicated. The green and blue dotted lined area represent for hypoxic and normoxic tumor area, respectively. Scale bar: 50 µm.
