# Supplementary material for: Hypoxic colorectal cancer cells promote metastasis of normoxic cancer cells depending on IL-8/p65 signaling pathway
Source: Cell Death Dis. 2020 Jul 31;11(7):610. doi: 10.1038/s41419-020-02797-z (PMC7395770; doi:10.1038/s41419-020-02797-z)
Supplement: Supplementary file 7 — Supplementary Information [file 41419_2020_2797_MOESM7_ESM.docx]

Supplementary Information for

Hypoxic colorectal cancer cells promote metastasis of normoxic cancer cells depending on IL-8/p65 signaling pathway

Yulong Mi1,2*, Lei Mu1,2*, Kaiyu Huang1,2, Yibing Hu1,2, Chang Yan1,2, Hui Zhao2, Chensen Ma1,2, Xiaolan Li2, Deding Tao2, Jichao Qin1,2^#^

*These authors contributed equally to this work.

^#^ Jichao Qin, MD, PhD

Email: [jcqin@tjh.tjmu.edu.cn](mailto:jcqin@tjh.tjmu.edu.cn)

**Supplementary Information**

1. **Materials**

|  | **Company** | **Cat.#** | **Dilution** |
| --- | --- | --- | --- |
| **Immunoblotting**  HIF1alfa  IL8  p65  phospho-p65  NF-kappa B pathway sample kit  E-cadherin  Vimentin  GFP  beta-actin | Cell Signaling Technology  R&D Systems  Cell Signaling Technology  Cell Signaling Technology  Cell Signaling Technology  Cell Signaling Technology  Cell Signaling Technology  Cell Signaling Technology  Abcam | 36169  MAB208-100  8242  3033  9936  3195  5741  2956  ab6276 | 1:1000  1:500  1:1000  1:1000  1:1000  1:1000  1:1000  1:1000  1:1000 |
| **Immunofluorescence**  HIF1alfa  IL8  GFP  CD31  Goat Anti-Mouse IgG (H+L), Alexa Fluor 488  Goat Anti-Rabbit IgG (H+L), Alexa Fluor 488  Goat Anti-Rabbit IgG (H+L), Cy3  Anti-Rabbit IgG(H+L), Alexa Flour 647 | Cell Signaling Technology  R&D Systems  Cell Signaling Technology  Beyotime Biotechnology  Jackson Immuno Research  Jackson Immuno Research  Jackson Immuno Research  Cell Signaling Technology | 36169  MAB208-100  2956  AF0099  115-545-003  111-545-003  111-165-003  4414 | 1:1000  1:500  1:75  1:200  1:50  1:50  1:50  1:500 |
| **Immunohistochemistry**  CA9 | GeneTex | GTX70020 | 1:200 |

**Table S1.** Antibodies used during experimental procedures.

|  | **Genes** |  | **Oligo sequences (5’ to 3’)** |
| --- | --- | --- | --- |
| **PCR** | *EGFP* | forward | GAAGAACGGCATCAAGGTG |
|  |  | reverse | CTCCAGCAGGACCATGTGA |
|  | *β-actin* | forward | GCAAGCAGGAGTATGACGAG |
|  |  | reverse | TCCACCACCCTGTTGCTGTA |
| **RT-qPCR** | *IL-8* | forward | ACACTGCGCCAACACAGAAATTA |
|  |  | reverse | TTTGCTTGAAGTTTCACTGGCATC |
|  | *p65* | forward | GTGGGGACTACGACCTGAATG |
|  |  | reverse | GGGGCACGATTGTCAAAGATG |
|  | *MMP1* | forward | CCAGGCCCAGGTATTGGACGGG |
|  |  | reverse | CAAATAAAGCCATGCCAATC |
|  | *MMP2* | forward | ACCAGCTGGCCTAGTGATGATGT |
|  |  | reverse | GGGGCAGCCATAGAAGGTGTTCA |
|  | *MT1MMP* | forward | AAGCAGCAGCTTCAGCCCCG |
|  |  | reverse | GCAGCGATGGCCGCTGAGAG |
| **RNAi** | *IL-8* | target sequence | GATGCCAGTGAAACTTCAA |
|  |  | code | siB11125103058 |
|  | *p65* | Sense | GGACAUAUGAGACCUUCAA dTdT |
|  |  | Antisense | UUGAAGGUCUCAUAUGUCC dTdT |

**Table S2**. Sequences of the primers used for PCR, RT-qPCR and siRNA.

| **Patient No.** | **Age** | **Gender** | **TNM** | **Tumor Grading** |
| --- | --- | --- | --- | --- |
| **XhCRC** | 47 | Female | T4bNx(4/10)M0 | G3 |
| **CRC1** | 42 | Female | T2N1a(1/15)M0 | G3 |
| **CRC30** | 63 | Male | T3Nx(0/10)M0 | G2-G3 |
| **CRC32** | 48 | Male | T3N0(0/14)M0 | G2 |
| **CRC33** | 62 | Male | T3N0(0/14)M0 | G2 |
| **CRC34** | 50 | Male | T4aN2b(22/26)M0 | G3 |
| **CRC36** | 62 | Female | T3N0(0/17)M0 | G2 |
| **CRC44** | 68 | Male | T3Nx(0/4)M0 | G2-G3 |
| **CRC45** | 64 | Female | T3N2b(8/13)M0 | G3 |
| **CRC94** | 61 | Female | T4aN0(0/22)M0 | G2 |

**Table S3**. Clinical history of human subjects.

1. **Methods**

***2.1 Cell culture***

For xhCRC cells, fresh xenograft tumors tissue was mechanically dissociated with sterilized scalpels and scissors, afterwards incubated for 1–2 h in DMEM/F12 containing 1.5 mg/ml collagenase IV(Gibco), 20 µg/ml hyaluronidase (Sigma Aldrich, St. Louis, MO, USA), 500 U/ml penicillin, 500 mg/ml streptomycin, and 1.25 mg/ml amphotericin B at 37 °C. In the back of filtering through sterile 100-μm strainers to obtain single-cell suspensions, red blood cells were then eliminated with a hypo-osmotic red blood cell lysis buffer (BioLegend, San Diego, CA, USA). Isolated single cells of xenograft tumors tissue were stained with EpCAM and subjected to FACS (Aria II, BD Biosciences, San Jose, CA, USA) to purify EpCAM-positive tumor cells. The isolated XhCRC cells were cultured in DMEM (Gibco) with 10% FBS (Gibco) and incubated in an incubator at 37°C with 5% CO_2_ for short-time culture and further experiment in vitro.

***2.2 Maintaining of human CRC xenograft tumors***

XhCRC were maintained in four weeks-old female NOD/SCID mice. Fresh human tissues acquired from surgical specimens from primary human colorectal tumor patients were dissociated into single cells, and then injected in 50% Matrigel (v/v with phosphate-buffered saline (PBS), BD Biosciences) SC in female NOD/SCID mice. After the tumors matured, they were harvested, human CRC cells were purified, and the cells were injected SC in 50% Matrigel in female NOD/SCID mice. This process was repeated every generation (~ 2 months).

***2.3 Hypoxic treatment***

We introduced two common types of treatment [10, 11]. First, cells were initially cultured in DMEM with 1% FBS in a hypoxic incubator with 5% CO2, 94% N2 and 1% O2 for 3 days, then in DMEM with 10% FBS in the general incubator for another 3 days, which was repeated for 10 or more cycles. Second, cells were cultured in DMEM with 1% FBS and 300-400 μM CoCl2 under normoxic conditions for 24 h to obtain CoCl2-induced HSS CRC cells.

***2.4 Conditioned medium preparation***

CM was derived from HSS CRC cells or CoCl_2_-induced HSS CRC cells (CoCl_2_ CRC cells). After HSS CRC cells reached 90% confluency, cells were washed with PBS and incubated with fresh DMEM/F12 at 37 °C in hypoxic environment for 12 h to obtain HSS-CM. For CoCl_2_ CRC cells, after treated with CoCl_2_, cells were washed with PBS and incubated with fresh DMEM/F12 at 37 °C in normoxic environment for 2 h to obtain CoCl_2_-CM. CM was collected and centrifuged at 2000×g for 10 min at 4 °C. Supernatant was filtered through a 0.22-µm filter (Millipore, Billerica, MA, USA) to remove the cellular debris.

***2.5 Cell migration and invasion assays***

For wound healing assay, tumor cells were growing to 95% confluence in 6-well plates in DMEM without FBS overnight. And a 10 µl pipette tip was used to scratch the monolayer of cells. The cells were cultivated for 24 h, with DMEM/F12 as the control.

For transwell migration assay, tumor cells were suspended in 200 µl serum-free medium or the other specific media were seeded into the top chamber, and 800 μl medium containing 10% FBS were added to the bottom chamber.

For transwell invasion assay, the insert membranes were coated with Matrigel (50 μl/well, BD Biosciences) before adding the cells.

In Fig S2B, after 24 hours of culture, cells in the bottom chamber were collected and cell number was calculated by FACS. In Fig S2C, after 24 hours of culture, cells on the top side of PC membrane were removed by swab, those on the bottom side were pictured by fluorescence microscope, six visual fields were randomly chosen to calculate the number of migrated cells. In other transwell assay, after culturing 14 hours in migration assay and 24 hours in invasion assay, cells were then stained with 0.1% crystal violet for 30 minutes, and non-migrating or non-invading cells were removed. Six visual fields were randomly chosen to calculate the number of migrated cells. Each experiment was performed three times, relative fold is Test group/Control group ratio.

In transwell assay and wound healing assay, when we need to evaluate the cell numbers or closed gap, there will be two investigator, one who is not aware of the grouping is responsible for counting or measuring, and the other who is aware of the grouping is responsible for data statistics and analysis.

***2.6 In vivo mouse assays***

Mice were randomly divided into each group of each experiment (3-5 mice per group). The mice were all of the same age and were weighed to confirm that the weight difference was no more than 2 g. Then the mice were numbered according to the weight from light to heavy. For example, if we have 10 mice, any number starts from one number, we take 10 numbers from left to right from the random number table. They are arranged according to the animal number, and then sorted by the the random number from small to large. And the 1st, 3rd, 5th, 7th, 9th belong to group A, and the 2nd, 4th, 6th, 8th, 10th belong to group B.

For lung metastasis assay, tumor cells were re-suspended in 100 μl control medium and medium with specific treatment in each assay were injected into the mice via tail vein to observe the distant metastasis. In Fig 1D, after 8 weeks, all mice were sacrificed under general anesthesia after injection. All the suspicious lung metastasis sites were evaluated by histologic examination. In Fig 6D, considering mice bearing the subcutaneously implanted tumor, after 4 weeks, all mice were sacrificed under general anesthesia after injection. The lung metastasis are micrometastasis, so continuous sections of lung tissue were pictured by microscope, and the number of micrometastasis in each lung tissue was calculated. In other lung metastasis assays, LoVo-Luciferase cells were used, after 8 weeks, the mice were anesthetized by general anesthesia, intraperitoneally injected with 100ul luciferase substrates (30mg/ml, Promega), and placed into the imaging dark box platform to get the whole-body image, and the lung metastasis were evaluated by analyzing the photon flux.

For subcutaneously implanted tumor model, xhCRC cells infected with the HRE-GFP lentivirus (XhCRC HRE-GFP cells) in 100 μl PBS mixed with Matrigel at 1:1 ratio were subcutaneously implanted into 4-week-old female NOD/SCID mice. After the tumor size up to ~ 30 mm^3^, intratumoral injection with bevacizumab (MedChemExpress, Shanghai, China) was performed 1 time per 3 days to induce tumor hypoxia. A week later, tumor was harvested and the hypoxic condition was detected by the expression GFP and/or HIF 1α.

At the end point of the experiment, all survived mice should be included in the analysis. And there is no exclusion criteria.

For animal studys, there will be two investigator, one who is not aware of the reagents of the treatment is responsible for give the treatment and take the data, the other who is aware of the reagents of the treatment is responsible for data analysis.

***2.7 Immunoblotting***

Cells were lysed in NP40 lysis buffer with complete protease and phosphatase inhibitor cocktails (Sigma-Aldrich). And protein concentration was determined using BCA assay (Thermo Fisher Scientific). The procedures of SDS-PAGE and immunoblotting were described as our previous studies. A total of 20 μg of protein per sample was loaded onto SDS-PAGE gels and then transferred onto 0.22-μm polyvinylidene difluoride membranes (Millipore). Membranes were blocked with 5% BSA in Tris-buffered saline for 1 h and incubated with primary antibodies at 4°C for overnight, following by incubation with the horseradish peroxidase–conjugated secondary antibody at room temperature. Finally, the membranes were visualized with Thermo Pierce chemiluminescent (ECL) Western Blotting Substrate (Thermo Fisher Scientific) using a Tanon 5200 system.

For immunoblotting of proteins extracted from xenograft tissue, frozen samples were ground using a mortar and pestle, and resuspended in lysis buffer (NaCl 150 mM, Tris-HCl pH 7.5 10 mM, EDTA 1 mM, NP-40 1%, EGTA 1 mM, sodium fluoride 50 mM, β- glycerophosphate 40 mM, sodium pyrophosphate 10 mM, sodium vanadate 1 mM, PMSF 8mM, 1x Halt phosphatase inhibitor). Homogenized samples were sonicated for 1min, incubated for 10min at 4°C with agitation, and then cleared by centrifugation at 13000rpm for 15min. Subsequent processing proceeded as described above.

***2.8 RNA expression analysis***

For PCR, genomic DNA was purified from uninfected and infected xhCRC cells using the Universal Genomic DNA Extraction Kit (TaKaRa, Shiga, Japan). RNA was extracted using Trizol (TaKaRa) according to the manufacturer’s protocol, and cDNA was synthesized using PrimeScript RT Master Mix (TaKaRa). qPCR was performed using SYBR green reagents (TaKaRa) on an ABI PRISM 7300 Sequence Detection System (Applied Biosystems, Foster City, CA, USA). Expression data were uniformly normalized to the internal control Actin and the relative expression levels were evaluated using the ΔΔCt method. Primer sequences used are listed in Supplementary Experimental Procedures Table S2.

***2.9 RNA interference***

IL8 siRNA and p65 siRNA (RIBOBIO, Shanghai, China) at a final concentration of 50 nmol/L were transfected into CRCs with Lipofectamine 2000 reagent (Invitrogen) according to the manufacturer's instructions. Cells were collected for further assay at 48 hours after transfection. The sense and antisense of each siRNA are listed in the Supplementary Experimental Procedures Table S2.

***2.10 Immunofluorescence***

Immunofluorescence staining was performed in cultured cells, frozen or paraffin-embedded CRC specimens and xhCRC specimens. CRC cells were cultured in glass-bottomed Petri dishes in a monolayer overnight and subsequently fixed with 4% paraformaldehyde (PFA) for 10 min at room temperature. For frozen specimens, after being fixed with 4% PFA, the tissue was placed into Tissue-Tek OCT (SaKura Finetek USA) and sectioned into 8 μm slices. After being permeabilized with 0.025% Triton X-100 for 10 min, cells or tissues were blocked with 5% bovine serum albumin (BSA) in PBS for 1 h at room temperature. Primary antibodies were diluted in 5% BSA. After being incubated overnight at 4 °C, cells were rinsed with PBS and incubated with a fluorophore-conjugated secondary antibody (diluted 1:50 in 5% BSA) for 2 h at room temperature. Samples were stained with 4’,6-diamidino-2-phenylindole (DAPI; Sigma Aldrich) and mounted with Anti-fade mountant (Thermo Fisher Scientific) before being visualized via ﬂuorescence microscopy (Olympus BX53 or CKX41) or confocal microscope (Olympus FV1000).

***2.11 Immunohistochemistry***

Immunohistochemistry (IHC) was performed as previously described. Briefly, tumors embedded in paraffin blocks were deparaffinized, and hydrated through an ethanol series. After microwave antigen retrieval in DakoCytomation target retrieval solution pH 6 (Dako), slides were incubated in 0.3% hydrogen peroxide solution in methanol for 15 min at room temperature to inhibit internal peroxidase activity. Next samples were blocked with serum-free protein block solution (Dako) and incubated with corresponding primary antibodies overnight at 4°C. Next day slides were stained with EnVision+ System–HRP labeled Polymer (Dako) and visualized with DAB peroxidase substrate kit (Vector Laboratories). Semi-quantitative analysis was performed with Image Pro Plus to identify the density (IOD/Area).

***2.12 Cytokine Array and ELISA***

Cytokines were determined using a Human XL Cytokine Array Kit (ARY022, R&D Systems, Minneapolis, MN USA), and concentration of IL8 was determined using an ELISA kit (VAL103, R&D System), which is according to the manufacturer’s instructions.

***2.13 FACS***

FACS was performed according to the manufacturer’s instructions using a FACS Aria II Cell Sorter (BD Biosciences), followed by flow cytometric analysis using Diva software (BD Biosciences).

***2.14 Human subjects***

All human subjects studies were performed under the guidelines and protocols approved by the ethical committe of Tongji Hospital, Tongji Medical College, HUST (IRB ID: 20141106). And informed consent was obtained from all subjects.
